# Changing Food Consumption Patterns and Land Requirements for Food in the Six Geopolitical Zones in Nigeria

**DOI:** 10.3390/foods11020150

**Published:** 2022-01-06

**Authors:** Jeffrey Chiwuikem Chiaka, Lin Zhen, Yu Xiao

**Affiliations:** 1 Key Laboratory for Resources Use and Environmental Remediation, Institute of Geographic Sciences and Natural Resources Research, Chinese Academy of Sciences, Beijing 100101, China; cjchiwuikem2019@igsnrr.ac.cn (J.C.C.); xiaoy@igsnrr.ac.cn (Y.X.); 2 College of Resources and Environment, University of Chinese Academy of Sciences, Beijing 100049, China; 3 Anambra-Imo River Basin Development Authority, Agbala 460109, Imo State, Nigeria

**Keywords:** food quantity, food expenditure, food intake, household size, population, Geopolitical Zones in Nigeria

## Abstract

Research on food consumption in Nigeria has mainly focused on food intake, household diversity, and purchasing power. We investigated a knowledge gap for food consumed by households and the land requirements for food resulting from household consumption patterns. The food consumed and the household size determine the land requirement for food. Therefore, a quantity-based analysis and a land demand methodology were applied to derive household food quantity and land requirements for food respectively. The results show that a greater percentage of household income is spent on cereals and starchy roots as the main source of calories and that cowpea is a secondary food option for households. In addition, households are changing their dietary intake from rice to maize and rice to cassava and yams as a cheaper alternative and experts’ measurements of food security at the household level indicates that households in our study are moderately food insecure. Other findings show that the country’s specific and per capita land requirements for food have gradually increased between 2000 and 2018. Across the six geopolitical zones, Northern regions with higher populations have high land requirements for food, especially for rice and maize (cereals), while Southern regions have high land requirements for cassava and yams (starchy roots) due to their respective consumption and household sizes. In addition, from our study, the land requirements for food show the actual cropland area of South South fed 5000 households. Consequently, a scenario analysis shows that the land requirements for food in our study exceeds the entire geographical area of Nigeria. Therefore, continued population growth without improved living standards and adequate food production output per hectare will further exacerbate food insecurity and land shortage in Nigeria.

## 1. Introduction

The problem of hunger, poverty and malnutrition in vulnerable countries has continued to attract the attention of various international institutions and humanitarian agencies such as the United Nations World Food Programme (WFP). Sustainable food consumption and production, hunger and poverty reduction are important targets in the United Nations Sustainable Development Goals (SDGs) to be achieved by 2030 [1], and less than a decade before the target, the results so far in many countries are not encouraging [2,3]. The need to address the above issues stems from expert reports and studies which highlighted the global malnutrition burden and risk of hunger as a result of the overarching effect of low calorie consumption, low self-sufficiency and a lack of food diversity seen especially in developing countries, which are exacerbated by climate change, low income, low food production and lack of food access [4,5]. In addition, the advent of COVID-19, which has disrupted the global and national food supply chain, has added to the list of contributing factors to food insecurity and hunger [2,6,7] and these problems are linked to mortality and morbidity, especially among children. Various studies have shown that sub-Saharan Africa and South Asia face this challenge due to the high proportion of people living below the international poverty line [8,9,10,11]. However, South Asia is making progress in improving income and calorie consumption [12], while African governments have been slow to take action to reduce the burden of malnutrition and promote food security in their respective countries, despite their pledge to promote food security and poverty reduction by signing the Maputo Declaration on Agriculture and Food Security in 2003 [13]. As a result, researchers describe their progress as insufficient and suboptimal [11,14]. This is of great concern as Africa’s population is expected to double by 2050 [15] and population growth is a critical determinant of a country’s or region’s food security. Furthermore, Africa has an estimated 52% of the world’s remaining arable land, but this is not evenly distributed among countries in the region [16]. Therefore, the need to assess the amount of food consumption and to use the land appropriately for sustainable food production is a step in the right direction. Nevertheless, one in three people worldwide suffers from at least one form of malnutrition [17].

Consumption of staple foods such as rice, maize and cassava is very high in low- to middle-income countries [14,18,19,20], while middle- to higher-income countries are consuming fewer staple foods and changing their consumption patterns towards more meat, processed foods and dairy products [14,21,22,23]. However, changes in dietary patterns are influenced by per capita income, lifestyle/preferences, urbanization and economic development [14,24,25,26,27,28,29,30,31], resulting in a high demand for land resources [30] to cope with the diverse food consumptions. Such changes in dietary patterns are also known to increase greenhouse gas emissions, impact the climate, contribute to unbalanced dietary intake and health-related diseases, as well as further increasing pressure on ecosystem carrying capacity and land degradation [32,33,34,35,36,37,38]. Besides, the total land required for food is determined by the type of food, the quantities consumed and the size of the population [39]. Available studies state that the type of food determines the land requirement for food [39] more than the quantities of food consumed per capita [40], while others assume that the land requirement for food also depends on the yield per hectare [41]. An example of this is the fact that an average Indian diet requires less land than an average diet in the United States of America, despite the size of the population [40]. 

Land requirements for food have been analyzed at global, regional [42,43], country and rural levels [41,44,45,46,47] using models and thought experiments such as ALBIO (Agricultural Land use and BIOmass), the Human Appropriation of Land for Food (HALF) Index, questionnaires, household food consumption surveys, data on population, dietary habits and crop yields. Their studies concluded that our food choices affect the land used for agriculture, i.e. the land required for food depends on the type of food and the rate of change in food consumption patterns. However, this interaction is causing biodiversity to decline at an accelerating rate.

Against this background, Nigeria, the most populous country in Africa, is considered as a case study. This is significant because Nigeria ranks 98th out of 107 countries on the 2020 Global Hunger Index, has the second highest proportion of stunted children in the world [48], and an estimated 87 million people (41%) live below the international poverty line [49]. These suggest many households in the country are undernourished, facing socio-economic challenges and that land for food production for the teeming population may be limited. In addition, there are few studies on food consumption in Nigeria, and most focus on nutrient deficiencies, household diversity, overweight and obesity, and purchasing power [33,50]. Hence, there is a lack of research on food consumption in Nigeria in terms of quantity, i.e. food available per household in Nigeria by weight and land requirements for food based on their consumption pattern. Therefore, the novelty of this research is to fill the knowledge gap on food consumption patterns and LRF in Nigeria. This has prompted the study to estimate land requirements based on food consumption patterns. This is important as it enables effective land resource management policies and contributes significantly to the achievement of food security and sustainable production. 

Our objective is therefore to analyze the temporal and spatial patterns of food consumption and the factors influencing them, and to determine the land requirements for food in the six geopolitical zones of Nigeria by combining available data on household food expenditure, selected food prices and local agricultural data. In this way, the food gap in the country and an approach to estimate how much land is currently used to feed the current population can be determined, as well as providing an outlook on the land requirements for food in the event of population growth in the future in order to initiate measures for sustainable food production.

This paper is divided as follows: The study area is presented, which gives us an overview of Nigeria. The second section deals with the methodology and datasets. The third section presents the results of our quantity analysis and land requirements for food. The fourth and fifth sections focus on the discussion and conclusion respectively. Finally, the limitations of the study are discussed.

## 2. Material and Methods

### 2.1. Brief Introduction of the Study Area

**Figure 1:**
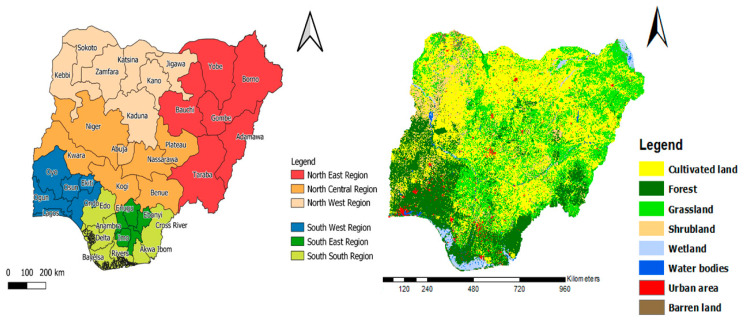
Location and land use land cover map of the six geopolitical zones in Nigeria.

Nigeria is a multicultural country with 36 states divided into six zones based on a number of criteria, including; cultures, ethnic composition, and a common history [51] namely: North Central, North East, North West, South East, South South, and South West. Based on remotely sensed data, 8 land use land cover types were identified (see Figure 1) in the country and being an agrarian country, it has a total arable land area estimated to be about 70.8 million ha, where about 43 million ha were under cultivation, with a low incidence of irrigation farming [52]. Nigeria’s climate permits the cultivation of a variety of crops and the Northern area which experiences sparse rainfall cultivates more of grains such as rice, millet, cowpeas, guinea corn, maize, and yams while the Southern areas grows more roots and tubers such as cassava, plantain, yams, and cocoyam in addition to oil palm, maize and rice [53] (see Table 1). Consequently, it has been observed that these frequently consumed traditional dietary foods are not enough to feed Nigerians [54]. Of the estimated population in 2016, the North has 54% (104,458,581) and South 46% (89,041,962). More than 70% of the Nigerian adult population is engaged in subsistence agriculture, which provides 90% of Nigeria’s agricultural products, and the majority live in rural areas [55]. Nigeria is a low to a middle-income country [56], and according to a poverty index survey by the World Bank, the North has poorer households (75%) compared to the South (25%) [57]. 

### 2.2. Data Source

In this study, the food balance sheet and production data of the Food and Agricultural Organization from 2000 to 2018 were used [58], including the General Household Survey in Nigeria (Living Standard Measurement Survey Wave 4) 2018–2019 [59], Consumption Expenditure Pattern in Nigeria [60], and Selected Food Price list from the Nigeria National Bureau of Statistics [61]. The GHS has a national sample size of approximately 5000 participating households at both urban and rural levels. To obtain an overview of the level of food consumption in Nigeria, the FAO (FAOSTAT) data for Nigeria from 2000 to 2018 were analyzed. However, due to the inconsistency and lack of data on cowpea in the FAO data, it was removed from the analysis of the baseline data.

Data on 2017 local food production and harvested area in the various states of Nigeria’s six geopolitical zones were compiled by the Federal Ministry of Agriculture and Rural Development (FMARD) [62] and the National Agricultural Extension and Research Liaison Services—an arm of the FMARD [63]. We assumed that crop production conditions and harvested areas remained the same for our analysis, as the years are not far apart.

Land use land cover (30 m) data from the Globeland30 project of the National Geomatics Center of China [64] were acquired to define the LULC pattern using ArcGIS 10.6 software (see Table 2).

### 2.3. Calculation of Household Food Consumption and Associated Land Requirement

The food quantity consumed by households in the six geopolitical zones was estimated from their mean household food expenditure calculated from the General Household Survey and Food Prices. To obtain the specific household food expenditure on a particular food item for the various geopolitical zones, the household food expenditure as obtained from the general household survey was multiplied by the percentage share of the national food expenditure of the same food item derived from the Consumption Expenditure Pattern in Nigeria. The reason for this calculation is to be more accurate as the GHS recorded the household food expenditures as food group aggregates (i.e., grains and flours) instead of individual food item expenditures (i.e., rice). Furthermore, the GHS mean household food expenditure data were a 7-day recall period. Therefore, the household food quantity was estimated for a year. However, due to a lack of data at the household level, the food groups under consideration to ascertain its quantity consumed based on average food expenditure and food prices in the six geopolitical zones are: Rice (*Oryza sativa*), Maize (*Zea mays*) (cereals), Cassava (*Manihot esculenta*), Yam (*Dioscorea villosa*) (starchy roots, tubers), Cowpea (*Vigna unguiculata*) (pulses), Onions (*Allium cepa*), and Tomatoes (*Solanum iycopersicum*) (vegetables), which are considered the traditional food system in Nigeria. Methods for analyzing these data and information are presented below.

(i) **Measuring household food quantity consumption.**

To determine the per annum quantity of household food consumption from their mean food expenditure in the six geopolitical zones, we applied the equation below:

(1) $$\overset{Expenditure}{\underset{fj=1\ldots 6\;geopolzone}{\prod }}\;\;=\sum \;\left(\mathrm{household}\;\mathrm{food}\;\mathrm{expenditure}\;\left(f\right)\;x\;\%\;\mathrm{share}\;\mathrm{in}\;\mathrm{food}\;\mathrm{consumption}\;\mathrm{expenditure}\;\mathrm{pattern}\;\mathrm{in}\;\mathrm{Nigeria}\right)$$

Therefore, to estimate the per annum household food quantity consumption, we used:

(2) $$Q_{\mathrm{fh}}=\sum \left(\frac{\prod_{fj=1\ldots 6\;geopolzone}^{Expenditure}\;\;\;}{Pf\;}\right)\;\times \;365\;\mathrm{days}$$

where: 

$\overset{Expenditure}{\underset{fj=1\ldots 6\;geopolzone}{\prod }}\;$ = food expenditure on food commodity (*f*) consumed by household (*j*) in the six geopolitical zones in Nigeria, 

**Q***_fh_* = the per annum quantity of food consumption per household in the six geopolitical zones,

**P***_f_* = the price per kg of the individual food items (*f*) in the six geopolitical zones in Nigeria.

Lastly, a Pearson correlation coefficient function was used to establish a relationship between household sizes and food expenditures using IBM SPSS 23.

(ii) **Estimating land requirements for food.**

The estimation of land requirements for food was analyzed in two parts, at the national level and in the six geopolitical zones, following a systematic approach.

**Part 1: LRF at the national level.**

LRF at the national level from 2000 to 2018 was estimated from major food consumption quantity (kg/capita/year) of rice, maize, cassava, yam, onions, and tomatoes from the FAOSTAT production dataset. However, due to the paucity of cowpea data from the FAO dataset, it was removed from the baseline analysis. 

**The steps involved in estimating LRF at the national level:**

**Figure 2:**
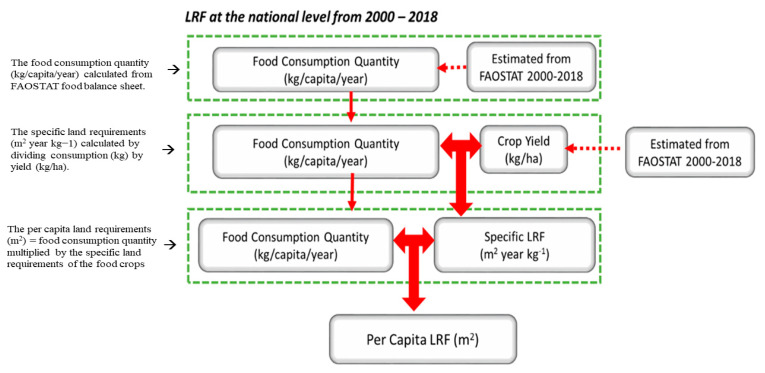
Diagram illustrating land requirements for food calculation at the national level.

(a) The food consumption quantity (kg/capita/year) of rice, maize, cassava, yam, onions, and tomatoes was calculated from the FAOSTAT food balance sheet from 2000 to 2018.
(b) The specific land requirements (m^2^ year kg^−1^) for each food considered in our research were calculated by dividing consumption (kg) in (a) by yield (kg/ha) collated from FAOSTAT from 2000 to 2018.
(c) The per capita land requirements (m^2^) for food were also calculated by combining the food consumption results in (a) and the specific land requirements of the food crops in (b) (see Figure 2).

**Part 2: LRF in the six geopolitical zones.**

LRF at the household level was estimated using the per annum household food quantity consumption from Equation (2) above, for rice, maize, cassava, yam, cowpea, onions, and tomatoes, and crop yield from the crop production and harvested area of 2017 in the 36 states therein grouped into the 6 geopolitical zones for the 2018–2019 analysis. 

In the analysis of household LRF, yield data were not available in some states for some of the crops examined in this study. Therefore, we used the national average yield value for 2017 from the FAO crop production data as a proxy, with the assumption that the soil conditions for the yield of the proxy crop are the same in the few states for which yield data were not available.

**The steps involved in estimating LRF in the six geopolitical zones:**

**Figure 3:**
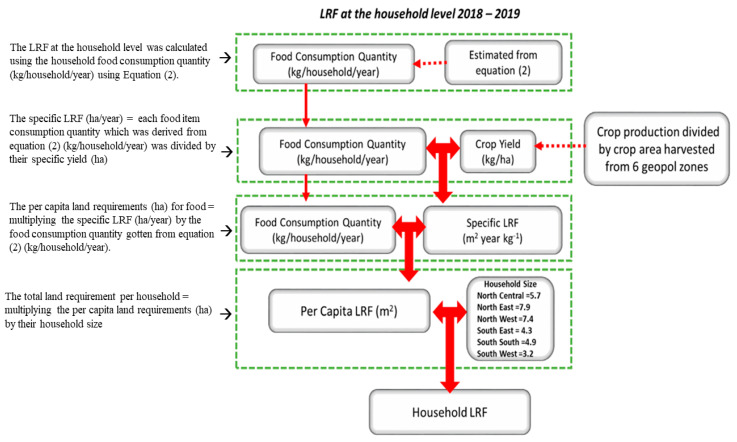
Diagram illustrating land requirements for food calculation in the six geopolitical zones.

(a) The LRF at the household level was calculated using the household food consumption quantity (kg/household/year) of rice, maize, cassava, yam, cowpea, onions, and tomatoes from Equation (2) above.
(b) To estimate the yield (kg/ha) as it is needed to calculate the LRF in the six geopolitical zones, the 36 states were grouped into their respective geopolitical zones and their average crop production and harvested area of rice, maize, cassava, yam, cowpea, onions, and tomatoes were calculated.
(c) To calculate the specific land requirements for food (ha/year), the 36 states were grouped into their respective geopolitical zones and each food item consumption quantity in (a) which was derived from Equation (2) (kg/household/year) was divided by their specific yield (ha) results obtained in (b).
(d) The per capita land requirements (ha) for food in the six geopolitical zones were calculated by multiplying the specific LRF (ha/year) in (c) by the food consumption quantity from Equation (2) (kg/household/year).
(e) The total land requirement per household in the six geopolitical zones was calculated by multiplying the per capita land requirements (ha) in (d) by their corresponding household size; that is, North Central (5.7), North East (7.9), North West (7.4), South East (4.3), South South (4.9), and South West (3.2) (see Figure 3).

## 3. Results

### 3.1. Food Consumption Patterns

#### 3.1.1. Food Consumption Patterns at the National Level

The summary of results as calculated by the FAO food balance sheets indicated that Nigeria’s total food consumption gradually increased from about 260 kg/capita in 2000 to 346.3 kg/capita in 2018, peaking in 2014. Cassava and yam dominated food consumption, followed by rice, maize, tomatoes, and onions, respectively. 

**Figure 4:**
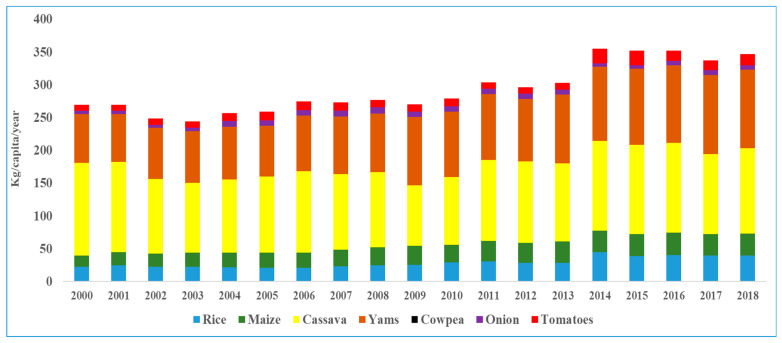
Nigeria food consumption pattern (kg/capita/year) from 2000 to 2018 (FAOSTAT).

Rice, which is the most popular staple, increased from 21.96 kg/capita in 2000 to 39 kg/capita in 2018, while maize showed a significant increased from 17.71 kg/capita in 2000 to 33.7 kg/capita in 2018 (see Figure 4). In as much as the cassava consumption pattern trend was significant, the consumption quantity declined from 140 kg in 2000 to 130.19 kg in 2018. Yam and tomato showed significant per capita increments (74–119 and 9.16–16.61 kg/capita, respectively), while onion consumption was 4.83 kg/capita in 2000 and 6.75 kg/capita in 2018 (see Figure 4).

#### 3.1.2. Food Consumption Patterns in the Six Geopolitical Zones

Of the six geopolitical zones, the North West had the highest per capita consumption of rice (137.81 kg/capita/year), followed by North East (112.1 kg/capita/year) and North Central (90.05 kg/capita/year), while the South West consumed 56.71 kg/capita/year, South East 40.84 kg/capita/year, and South South 31.25 kg/capita/year, respectively. 

Similarly, per capita maize consumption is also high in the North, as the North East consumed 186.04 kg/capita/year, North West 139.49 kg/capita/year, and North Central 59.54 kg/capita/year. Likewise, in maize consumption, the South West remains the highest per capita consumer of maize in the South, with 31.27 kg/capita/year, followed by South East 8.48 kg/capita/year and South South 4.43 kg/capita/year. The high consumption of rice and maize thus shows that cereals are an important staple food for people in the North (Table 3).

The per capita quantity of cassava consumption shows that households in the South South consumed more cassava (199.62 kg/capita/year), followed by South East (159.66 kg/capita/year), North Central (88.96 kg/capita/year), and South West (65.45 kg/capita/year), while households with low per capita consumption of cassava were in the North East (17.88 kg/capita/year) and North West (13.98 kg/capita/year).

Yam consumption was highest in the South East (158.99 kg/capita/year), South South (132.04 kg/capita/year), and North Central (68.23 kg/capita/year), while it was lower in the South West (49.85 kg/capita/year), North East (22.74 kg/capita/year), and North West (12.55 kg/capita/year). 

The total quantity of cowpea consumption was higher in the North than in the South. However, by per capita consumption, the North East is the highest (18.36 kg/capita/year), closely followed by South South (16.13 kg/capita/year), North Central (15.02 kg/capita/year), South East (13.09 kg/capita/year), North West (11.36 kg/capita/year), and South West (12.21 kg/capita/year).

**Figure 5:**
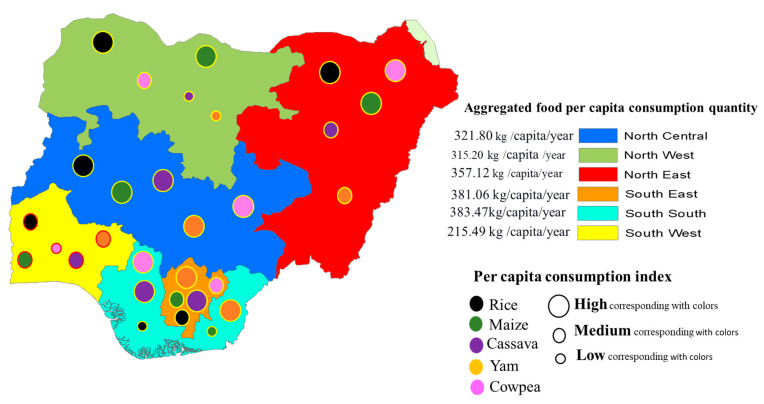
Pictorial representation of per annum household food consumption distribution and the rate of consumption of staple food (excluding onion and tomato) in the six geopolitical zones.

Summarizing the quantities of staple foods consumed (excluding onions and tomatoes) by geopolitical zones in Nigeria, North East and North West had the highest total food quantity consumption of 2821.22 and 2332.44 kg/year, respectively, albeit mainly from cereals. Following behind are South South (1878.99 kg/year), North Central (1834.28 kg/year), South East (1638.58 kg/year), and South West (689.58 kg/year). However, by per capita consumption derived from their household sizes, South South and South East consumed more food (383.47 and 381.06 kg/capita/year) respectively, followed by North East (357.12 kg/capita/year), North Central (321.80 kg/capita/year), North West (315.20 kg/capita/year), and finally, South West (215.49 kg/capita/year) (see Figure 5). Therefore the results from the food consumption quantity across the 6 geopolitical zones in Nigeria show that North Central had a 16.4% share of the total average, North East 25.2%, North West 20.8%, South East 14.6%, South South 16.8%, and South West 6.2%.

### 3.2. Analysis of Land Requirements for Food

#### 3.2.1. LRF at the National Level

The per capita land required for staple food was decoupled to observe the specific land requirements for the food considered in this study. From the FAO data, it shows that the per capita land requirements percentage increase of rice and maize was 231.7% and 192.1%, respectively, from 2000 to 2018. Cassava per capita LR decreased by 64.7% from 2000 to 2009, and later increased by 180.2% from 2009 to 2018. Other food items’ land requirements, such as yam, onions, and tomato, increased by 249.4%, 280%, and 76.7%, respectively, from 2000 to 2018.

#### 3.2.2. Land Requirements for Food in the Six Geopolitical Zones

The specific and household land requirements for food varied across the six geopolitical zones (see Table 4). Rice, a popular staple in Nigeria, had a high per capita demand for land, especially in the North. Rice per capita land requirement was 10.20 ha/capita/year in North West, 8.18 ha/capita/year in North East, and 3.06 ha/capita/year in North Central, while the South West had 0.79 ha/capita/year, South East 0.53 ha/capita/year, and South South 0.38 ha/capita/year. 

In addition, maize also has high per capita requirements in the North compared to the South. The North East per capita LR for maize was 19.35 ha/capita/year, North West 9.62 ha/capita/year, North Central 1.13 ha/capita/year, and their Southern counterparts have a low per capita land requirement for maize: South West 0.22 ha/capita/year, South South 0.1 ha/capita/year, and South East 0.03 ha/capita/year. 

In terms of starchy roots, the household land requirements for cassava show that the food is consumed more in the South South (17.59 ha/year), South East (6.19 ha/year), and North Central (4.56 ha/year), while it has low household land requirements in the South West (0.42 ha/year), North East (0.40 ha/year), and North West (0.22 ha/year) due to its lower consumption rate. Similarly, the household land requirements for yam are high in the South South (7.11 ha/year) and South East (5.46 ha/year), followed by North Central (1.14 ha/year), North East (0.87 ha/year), South West (0.32 ha/year), and North West (0.30 ha/year).

Cowpea, which serves as a major source of protein in most households, has a land requirement of 2.29 ha/year in the North East, which is the highest, followed by North Central (0.97 ha/year), South South (0.88 ha/year), North West (0.81 ha/year), South East (0.39 ha/year), and South West (0.22 ha/year).

Onion and tomato are vegetables used in daily cooking across the six geopolitical zones, and onion has higher household land requirements across the six geopolitical zones compared to tomato (see Table 4). 

Lastly, the household land requirement for food in the six geopolitical zones was 515.10 ha/year. Therefore, the percentage distribution across the country shows that North Central had 7.04%, North East 43.4%, North West 29.15%, South East 10.78%, South South 8.23%, and South West 1.4% of the total average of the household land requirements for food. 

## 4. Discussion

Food consumption patterns in the six geopolitical zones show that cereals and starchy roots contribute significantly (over 70%) to household calorie requirements. Available studies have shown that developing countries rely on starchy staple foods to meet their energy needs. For example, a similar observation was made in the city of Guyuan in Northwest China that the energy needs of the population are met by staple foods [44]. 

The quantity of household food consumption was estimated from household food expenditure and food prices. Therefore, the share of total household expenditure (as a proxy of income) spent on food is an indicator of household food security, and low-income households spend more of their income on providing food [65]. The Nigeria National Bureau of Statistics revealed that 59.19% was the total expenditure on food in 2019 by households in Nigeria [66]. This shows that many Nigerian households spend a substantial amount of their income on providing food, and according to experts’ measurements of food security at the household level [67], this classifies households in our study as being moderately food-insecure. Based on the aforementioned findings, the North East are more food insecure as they spent more to feed themselves having the highest percentage (25.2%) of the total average of food consumed in the six geopolitical zones, followed by the North West (20.8%), South South (16.8%), North Central (16.4%), South East (14.6%), and the least is the South West (6.2%).

The food consumption pattern results indicate that households are changing their dietary patterns due to their high food expenditures. This is observed in the North East and North West, indicating that the two zones are shifting their consumption patterns from rice to maize as a cheaper alternative, likewise in the South East and South South, consuming more dense calories from cassava and yam than rice and maize. Additionally, an interesting observation is that the South West consumes more cereals, especially rice, than its southern counterparts. A plausible reason for this is that the South West have more rice-producing states [68], which may have influenced the price of rice to be relatively cheaper than in other parts of the country. The North Central zones also consume more amount of cereals and starchy roots as they have the latitude to produce these crops [69].

Cowpea is one of the least consumed staple foods in terms of quantity in the six geopolitical zones, which suggests that most households prefer to consume other sources of protein, such as meat, fish, and eggs. However, cowpea serves as a good source of protein in both the North and the South. Aside direct consumption, cowpea are also used for cooking, as it can be used as a thickener [20], and popular local snacks (*akara* and *moi-moi*) are made from its derivatives. 

Onions and tomatoes are among the daily cooking ingredients throughout the country, and their consumption depends on the season.

One of the factors considered in our study that influences food consumption patterns is the different household sizes. The North has an average household size of 7, and the average for the South is 4, which further influences the quantity of food per household. To demonstrate the influence of household size on the amount of food consumed, we calculated the Pearson correlation coefficient, which showed a significant relationship of 0.636 between household size and food expenditure. 

**Figure A1:**
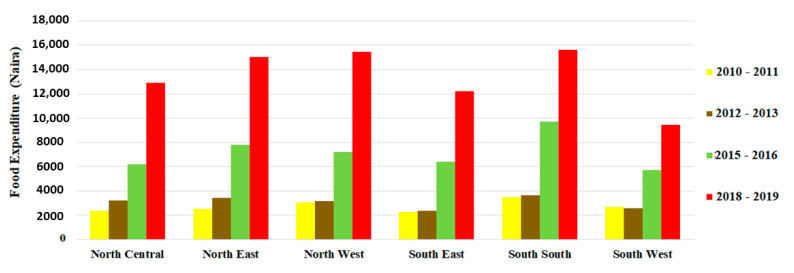
Food expenditure per week for grains and flours, starchy roots, tubers and plantains, pulses, nuts, and seeds, vegetables, meat, fish, and animal products for the 6 geopolitical zones from 2010 to 2019.

Another reason can be attributed to socio-economic aspects, such as low income and rising food prices that make it difficult to access food (see Figure A1). These factors affect the food demand and consumption pattern [70,71] as low income households tend to consume local-specific, affordable, and carbohydrate-rich food items. 

Apart from land holdings and suitability for crop cultivation, food culture also has a significant impact on food choices in the country and it may be difficult for households to change their consumption patterns [69]. For brevity, a culture of making a gelatinized semi-solid paste from these grains and tubers, which are consumed with locally prepared soups by households across the country, is widespread [20,55], and this is consumed more than once a day. This influence of food culture is also documented in various studies in China [38,72].

**Figure 6:**
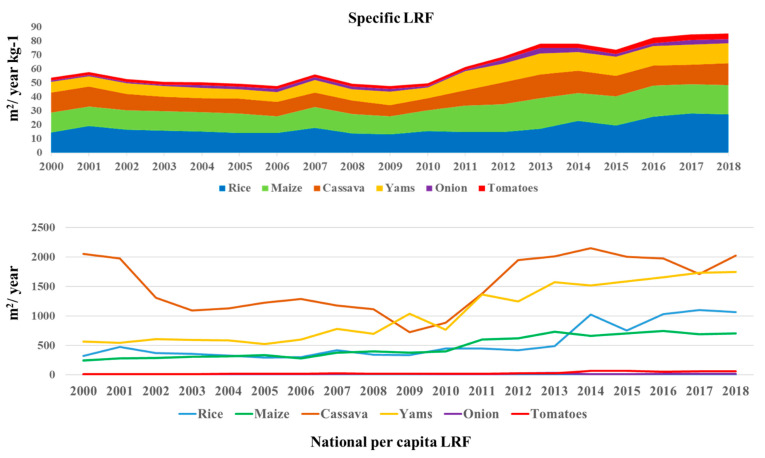
National specific and per capita land requirements for food in Nigeria (data source: FAOSTAT).

In terms of land required for food, the country’s specific land requirement for food has increased from 2000 to 2018 (Figure 6). This indicates that food demand and consumption is increasing for Africa’s largest population. Similar observations were made in a study in the Philippines on the increasing population raising land requirements for food [46]. The Northern zones having a larger household size and population translated to higher land requirements for the food quantity they consumed compared to the Southern zones. 

Rice, which is a popular staple in Nigeria, has one of the highest specific land requirements in the six geopolitical zones (Table 4). Likewise, in the Philippines in South Asia, rice was a major contributor to their land requirements for food [46]. Cereals and starchy roots are commonly consumed food groups in Nigeria, and therefore require more land based on their consumption. The North East and North West have the highest per capita land requirements for maize, which suggests that households in these zones are replacing rice consumption with maize, possibly to reduce the cost of food expenditure, as previously mentioned. 

Overall, the increases in land requirements from the study are attributed to food culture, income/expenditure, and population growth. In addition, two critical findings on the land requirements for food from the study showed that the actual cropland area of the South South zone (comprising of six states) was used to feed approximately 5000 households (study sample size), and more land, which exceeded the actual total land area of Nigeria, will be needed to feed approximately 40 million households (see Table 1 and Table 5). Therefore, considering a “business as usual” scenario on land requirements for food from our study, a further increase in population will pose a challenge to the actual arable land and its resources to accommodate the food consumption patterns.

## 5. Conclusions

The objective of this study was to analyze the food consumption patterns over time and space to identify their influencing factors and to determine the land requirements for food in the six geopolitical zones of Nigeria. From this, the following conclusions were drawn:

(i) From the food considered in our study, households depend on rice, maize, cassava, and yams for their daily calorie requirements. This dietary pattern, which comprises of excess consumption of starchy foods, may have health and nutritional consequences. In addition, cowpea is the least consumed, albeit serving as a major source of protein in the country, consumed more by the Northern households than the Southern households. The South South, South East, and North Central regions consume more starchy roots, and the South West maintained the least food quantity consumed.
(ii) The household food consumption quantity was derived from household food expenditure and its corresponding food prices. Therefore, the current consumption pattern indicates that larger households will be vulnerable to food price inflation and accessibility, which poses a risk to their food security and vulnerable to undernourishment, as their food expenditure, which is high in this study, will be higher if current conditions persist.
(iii) Many households are changing their food consumption patterns to cheaper alternatives, especially from rice to maize, and rice to cassava and yam, which suggests that rice is more expensive than other food items.
(iv) Cereals and starchy roots have high household land requirements, as they are commonly consumed food groups, and the North East (50.4%) and North West (31%) have the highest household land requirements for food, with the South West (0.7%) having the lowest. The driving factors include population, household sizes, income/expenditure, food culture/preference, and food prices.
(v) Food items constantly consumed by households have high land requirements across the six geopolitical zones, as seen for rice, maize, cassava, and yam, to satisfy their consumption patterns (see Table 4), irrespective of having low specific land requirements.

Finally, looking at the land requirements for food, a further increase in population without adequate food production output in the country, poses a challenge to the actual arable land and its resources to meet food consumption.

We recommend improving the standard of living of the people so that they have access to enough food for a healthy life, and making efforts to promote food diversity and improve food production to reduce the high cost of food expenditure. If expansion of cropland is considered, abandoned land can be converted to cropland and rapid urbanization and infrastructure development should take into account the need to preserve arable land for the growing population, because a future increase in the number of households without adequate food production and within the currently available cropland poses a serious challenge to food security in Nigeria.

### Limitations

The use of secondary data has its limitations. In the general household survey, households may overestimate or underestimate their food expenditure. In addition, the GHS recorded the cumulative expenditure on food rather than on a single food item. We had to use the percentage share of food expenditure for each item to obtain our values. This is because the GHS did not provide information on the percentage distribution of the household sample size of 5000 and the age of household members in each geopolitical zone. 

Due to the lack of recent data, the 2017 crop production and harvested area were used to estimate yield. However, for some crops, data were not available, and the national average was used. In addition, the FAO dataset contains estimated values and an inconsistent cowpea values at the national level that may have influenced the results.

The impact of the COVID-19 pandemic disrupted our preparations to travel to Nigeria for fieldwork and to obtain information on the field. Access to up-to-date national data was also a challenge. We therefore used secondary data from international organizations, Nigeria national bureau of statistics and Nigerian ministry in charge of agricultural data for this study.

Further studies are needed to include more food items to estimate their consumption quantities, determine their land requirements and propose sustainable food production techniques amidst present environmental boundaries.

## Figures and Tables

**Table 1:** The six geopolitical zones’ information at a glance.

Zones	Average Household Size (2018–2019)	Total Land Area (ha) (2020)	Cropland Area (ha) (2020)	Crops Commonly Cultivated and Farming System	Population (2016)	Ethnic Groups
North Central	5.7	24,242,500	12,230,953	Cassava, yams, sorghum, millet, maize, rice, cowpea, and commercial livestock farming.	29,252,408	Tiv, Igala, Berom, and others.
North East	7.9	27,239,500	11,880,402	Maize, millet, sorghum, wheat, rice, cowpea, and commercial livestock farming	26,263,866	Hausa
North West	7.4	21,606,500	13,271,431	Rice, millet, sorghum, maize, cowpea, and vegetables, and commercial livestock farming.	48,942,307	Hausa
South East	4.3	2,952,500	1,406,476	Cassava, yams, maize rice, and subsistence livestock farming	21,955,414	Igbo
South South	4.9	8,458,700	2,507,664	Cassava, yam, vegetables, plantain, and subsistence livestock farming	28,829,288	Ibibio, Bini, Ijaw, and others.
South West	3.2	7,966,500	2,442,536	Rice, maize, casava, yam, plantain, and subsistence livestock farming.	38,257,260	Yoruba
National	5.5	92,466,200	43,739,462	Mixed farming	193,500,543	Major: Igbo, Hausa and Yoruba

**Table 2:** Summary of the data source information.

Data Source	Data	Year
Food and Agricultural Organization (FAOSTAT)	Food Balance Sheet and Production	2000–2018, 2017
Nigeria Bureau of Statistics	General Household Survey in Nigeria (LSMS Wave 4)	2018–2019
Nigeria Bureau of Statistics	Consumption Expenditure Pattern in Nigeria	2019
Nigeria Bureau of Statistics	Selected Food Prices	2018
Federal Ministry of Agriculture and Rural Development (FMARD) and the National Agricultural Extension and Research Liaison Services (NAERLS)	Food Crop Production and Harvested Area Statistics	2017
National Geomatics Center of China	30 m land use land cover images	2020

**Table 3:** Total per annum household food quantity consumption in the six geopolitical zones.

Zones	Average Household Size	Combined Population (2016)	RICE (kg)	MAIZE (kg)	CASSAVA (kg)	YAM (kg)	COWPEA (kg)	ONION (kg)	TOMATO (kg)
North Central	**5.7**		513.30	339.37	507.10	388.93	85.59	160.66	146.79
North East	**7.9**	104,458,581	885.59	1469.69	141.22	179.66	145.06	225.81	186.61
North West	**7.4**		1019.82	1032.20	103.52	92.86	84.04	210.00	168.43
South East	**4.3**		175.61	36.48	686.55	683.66	56.28	222.25	186.56
South South	**4.9**	89,041,962	153.12	21.71	978.13	647.00	79.03	133.68	93.10
South West	**3.2**		181.49	100.07	209.44	159.52	39.07	113.48	99.62

**Table 4:** Land requirements per food item based on food quantity consumption in the 6 geopolitical zones.

Zones	Food Item	Specific Land Requirement (ha/Year kg^−1^)	Per Capita Food Consumption (kg/Capita/Year)	Total Household Food Consumption (kg Year^−1^)	Per Capita Land Requirement (ha/Year)	Household Land Requirement (ha/Year)	Household Sizes
**North Central**	Rice	0.034	90.05	513.30	3.06	17.44	
	Maize	0.019	59.54	339.37	1.13	6.44	
	Cassava	0.009	88.96	507.10	0.80	4.56	
	Yam	0.003	68.23	388.93	0.20	1.14	**5.7**
	Cowpea	0.011	15.02	85.59	0.17	0.97	
	Onion	0.032	28.19	160.66	0.90	5.13	
	Tomato	0.004	25.75	146.79	0.10	0.57	
	Sub-total	**0.112**	**375.74**	**2141.74**	**6.37**	**36.25**	
**North East**	Rice	0.073	112.10	885.59	8.18	64.62	
	Maize	0.104	186.04	1469.69	19.35	152.87	
	Cassava	0.003	17.88	141.22	0.05	0.40	
	Yam	0.005	22.74	179.66	0.11	0.87	**7.9**
	Cowpea	0.016	18.36	145.06	0.29	2.29	
	Onion	0.008	28.58	225.81	0.23	1.82	
	Tomato	0.004	23.62	186.61	0.09	0.71	
	Sub-total	**0.213**	**409.32**	**3233.64**	**28.32**	**223.57**	
**North West**	Rice	0.074	137.81	1019.82	10.20	75.48	
	Maize	0.069	139.49	1032.20	9.62	71.19	
	Cassava	0.002	13.99	103.52	0.03	0.22	
	Yam	0.003	12.55	92.86	0.04	0.30	**7.4**
	Cowpea	0.010	11.36	84.04	0.11	0.81	
	Onion	0.007	28.38	210.00	0.20	1.48	
	Tomato	0.004	22.76	168.43	0.09	0.67	
	Sub-total	**0.169**	**366.34**	**2710.87**	**20.29**	**150.15**	
**South East**	Rice	0.013	40.84	175.61	0.53	2.28	
	Maize	0.003	8.48	36.48	0.03	0.13	
	Cassava	0.009	159.66	686.55	1.44	6.19	
	Yam	0.008	158.99	683.66	1.27	5.46	**4.3**
	Cowpea	0.007	13.09	56.28	0.09	0.39	
	Onion	0.179	51.69	222.25	9.25	39.78	
	Tomato	0.007	43.39	186.56	0.30	1.29	
	Sub-total	**0.226**	**476.14**	**2047.39**	**12.91**	**55.51**	
**South South**	Rice	0.012	31.25	153.12	0.38	1.86	
	Maize	0.002	4.43	21.71	0.01	0.05	
	Cassava	0.018	199.62	978.13	3.59	17.59	
	Yam	0.011	132.04	647.00	1.45	7.11	**4.9**
	Cowpea	0.011	16.13	79.03	0.18	0.88	
	Onion	0.108	27.28	133.68	2.95	14.46	
	Tomato	0.005	19.00	93.10	0.09	0.44	
	Sub-total	**0.167**	**429.75**	**2105.77**	**8.65**	**42.39**	
**South West**	Rice	0.014	56.71	181.49	0.79	2.53	
	Maize	0.007	31.27	100.07	0.22	0.70	
	Cassava	0.003	65.45	209.44	0.13	0.42	
	Yam	0.002	49.85	159.52	0.10	0.32	**3.2**
	Cowpea	0.006	12.21	39.07	0.07	0.22	
	Onion	0.025	35.46	113.48	0.89	2.85	
	Tomato	0.002	31.13	99.62	0.06	0.19	
	Sub-total	**0.059**	**429.75**	**902.69**	**8.65**	**42.39**	
	**TOTAL**	**0.946**	**2487.04**	**13,142.1**	**85.19**	**515.1**	

**Table 5:** Current gap in household land requirements for food in Nigeria and in the six geopolitical zones.

Zones	Combined Household Land Requirements of the 7 Food Crops (Ha)	Projected Household Land Requirements of the 7 Food Crops (Ha)
North Central	36.25	2016 estimated population = 193 million ↓ National average household size of 5 ↓ Which gives 38.6 million households in Nigeria. ↓ 38.6 million households × our study household LR of 515.1 ha
North East	223.57	
North West	150.15	
South East	55.51	
South South	42.39	
South West	7.23	
TOTAL	**515.1**	
National GHS of 5000 households	**2,575,500**	**Gap = 19,882,860,000**

## Data Availability

Food Balance Sheet and Production https://www.fao.org/faostat/en/#data/FBS (accessed on 15 June 2020); General Household Survey in Nigeria (LSMS Wave 4) 2018–2019 https://www.nigerianstat.gov.ng/ (accessed on 25 June 2020); Consumption Expenditure Pattern in Nigeria 2019 https://nigerianstat.gov.ng (accessed on 8 June 2020); Selected Food Prices 2018 https://nigerianstat.gov.ng/ (accessed on 25 June 2020); Food Crop Production and Harvested Area Statistics 2017 https://fmard.gov.ng/ (accessed on 29 June 2021); 30 m land use land cover images 2020 http://www.globallandcover.com/ (accessed on 8 June 2020).
